# ANGPTL3 is a novel biomarker as it activates ERK/MAPK pathway in oral cancer

**DOI:** 10.1002/cam4.418

**Published:** 2015-01-30

**Authors:** Tomoyoshi Koyama, Katsunori Ogawara, Atsushi Kasamatsu, Atsushi Okamoto, Hiroki Kasama, Yasuyuki Minakawa, Ken Shimada, Hidetaka Yokoe, Masashi Shiiba, Hideki Tanzawa, Katsuhiro Uzawa

**Affiliations:** 1 Department of Oral Science, Graduate School of Medicine, Chiba University1-8-1 Inohana, Chuo-ku, Chiba, 260-8670, Japan; 2 Department of Dentistry and Oral-Maxillofacial Surgery, Chiba University Hospital1-8-1 Inohana, Chuo-ku, Chiba, 260-8670, Japan; 3 Department of Oral and Maxillofacial Surgery Research Institute, National Defense Medical College3-2 Namiki, Tokorozawa, Saitama, 359-8513, Japan; 4 Department of Clinical Oncology, Graduate School of Medicine, Chiba University1-8-1 Inohana, Chuo-ku, Chiba, 260-8670, Japan

**Keywords:** Angiopoietin-like 3, extracellular signal-regulated MAP Kinases, head and neck, squamous cell carcinoma, survival

## Abstract

Angiopoietin-like 3 (ANGPTL3), which is involved in new blood vessel growth and stimulation of mitogen-activated protein kinase (MAPK), is expressed aberrantly in several types of human cancers. However, little is known about the relevance of ANGPTL3 in the behavior of oral squamous cell carcinoma (OSCC). In this study, we evaluated ANGPTL3 mRNA and protein in OSCC-derived cell lines (*n* = 8) and primary OSCCs (*n* = 109) and assessed the effect of ANGPTL3 on the biology and function of OSCCs in vitro and in vivo. Significant (*P* < 0.05) ANGPTL3 upregulation was detected in the cell lines and most primary OSCCs (60%) compared with the normal counterparts. The ANGPTL3 expression level was correlated closely (*P* < 0.05) with tumoral size. In patients with T3/T4 tumors, the overall survival rate with an ANGPTL3-positive tumor was significantly (*P* < 0.05) lower than that of ANGPTL3-negative cases. In vitro, cellular growth in ANGPTL3 knockdown cells significantly (*P* < 0.05) decreased with inactivated extracellular regulated kinase (ERK) and cell-cycle arrest at the G1 phase resulting from upregulation of the cyclin-dependent kinase inhibitors, including p21^Cip1^ and p27^Kip1^. We also observed a marked (*P* < 0.05) reduction in the growth in ANGPTL3 knockdown-cell xenografts with decreased levels of phosphorylated ERK relative to control-cell xenografts. The current data indicated that ANGPTL3 may play a role in OSCCs via MAPK signaling cascades, making it a potentially useful diagnostic/therapeutic target for use in patients with OSCC.

## Introduction

Angiopoietin-like 3 (ANGPTL3) is a member of the angiopoietin-like proteins (ANGPTLs), which are functionally defined by the C-terminal fibrinogen-like domain that mediates binding to the Tie2 receptor and thereby facilitates a cascade of ultimate events regulating blood vessel formation 1–3. However, ANGPTL3 binds to integrins and not the Tie 2 receptor 1.

ANGPTL3 plays a vital role in regulating the plasma levels of triglyceride and cholesterol mainly via reversible inhibition of the lipoprotein lipase activity and vascular endothelial growth factors 2,3.

Previous studies have reported that ANGPTL3 activity is one of the most important factors in cancer growth and invasion 4, because of the mitogen-activated protein kinase (MAPK) signaling cascades 5. Growth of human cancer cells is controlled largely at the G1 phase of the cell cycle 6. ANGPLT3 was reported that specifically modulates upstream of the extracellular-regulated kinase (ERK)/MAPK signaling cascade by a previous study 7. The activation of ERK plays a fundamental role in G1/S transition 8,9. Protein/kinase inhibitory protein (Cip/Kip) family binds to cyclin–CDK complexes interacting to the members of the cyclin-dependent kinase and leads to G1 cell-cycle arrest. The levels of p21, p27, cyclin D1, and cyclin E are influenced by multiple signaling pathways including the (ERK)/MAPK signaling pathway 10–13. Based on this evidence, we hypothesized that ANGPTL3 may be associated with development of human cancer including oral squamous cell carcinoma (OSCCs).

This study shows the results of a comprehensive analysis of molecular/cellular subtypes of ANGPTL3 in OSCC that are linked clinically to tumoral progression and prognosis.

## Materials and Methods

### Ethics statement

The study protocol was approved by the Ethical Committee of the Graduate School of Medicine, Chiba University (approval number, 236) and performed in accordance with the tenets of the Declaration of Helsinki. All patients provided written informed consent before participating in this research.

The care of the animals was in accordance with the guidelines of Chiba University. The Committee on the Ethics of Animal Experiments of Chiba University approved the study protocol (approval number, 25–221).

### OSCC-derived cell lines and tissue specimens

Immortalized human OSCC-derived cell lines (Ho-1-N-1, Ho-1-u-1, HSC-2, HSC-3, HSC-4, Sa3, KOSC-2, and Ca9-22) were obtained from the Human Science Research Resources Bank (Osaka, Japan) or the RIKEN BioResource Center (Ibaraki, Japan) through the National Bio-Resource Project of the Ministry of Education, Culture, Sports, Science and Technology in Japan. Short tandem repeat profiles confirmed cellular identity. All OSCC-derived cell and primary cultured human normal oral keratinocytes (HNOKs) cultures were conducted as described previously 14–17.

### mRNA expression analysis

Real-time quantitative reverse transcriptase-polymerase chain reaction (qRT-PCR) was conducted as described previously 14–16. The information regarding primers were *ANGPTL3* (5′-CACTTCAACTGTCCAGAGGGTTA-3′ and 5′-GTTTTCTCCACACTCATCATGC-3′) and universal probe #75, and the glyceraldehyde-3-phosphate dehydrogenase (GAPDH) (5′-AGCCACATCGCTCAGACAC-3′ and 5′-GCCCAATACGACCAAATCC-3′) and universal probe #60.

### Immunoblotting analysis

Immunoblotting analysis was performed as described previously 14–17. The antibodies were anti-ANGPTL3 (Santa Cruz Biotechnology, Santa Cruz, CA), anti-GAPDH (Santa Cruz Biotechnology), anti-ERK1/2 (Cell Signaling Technology, Danvers, MA), anti-*α*-tubulin (Cell Signaling Technology), anti-phospho-ERK1/2 (Cell Signaling Technology), anti-p21^Cip1^ (Cell Signaling Technology), anti-p27^KIP1^ (Cell Signaling Technology), anti-cyclin D1 (Cell Signaling Technology), anti-cyclin E (Santa Cruz Biotechnology), anti-CDK2, anti-CDK4 (Cell Signaling Technology), or anti-CDK6 (Cell Signaling Technology).

### Immunohistochemistry

Immunohistochemistry and IHC scoring systems were performed as described previously 14–17. The antibodies were anti-ANGPTL3 (Santa Cruz Biotechnology). To calculate the 5-year survival rate, we surveyed each patient's life and month of death.

### Transfection with shRNA plasmid

Stable transfection was performed using Lipofectamine LTX and Plus Reagents (Invitrogen, Carlsbad, CA, USA), according to the manufacturer's instructions. ANGPTL3 shRNA (shANGPTL3) and the control shRNA (shMock) (Santa Cruz) vectors were transfected into HSC-3 and Sa3. After transfection, the cells were isolated by the culture medium containing 1 mg/mL Puromycin (Invitrogen). After 3–4 weeks, resistant cell colonies were picked and transferred to new dishes. ShANGPTL3- and shMock-transfected cells were used for further experiments.

### Cellular growth

Cellular growth assay was performed as described previously 14–17.

### Cell-cycle analysis

In order to synchronize cells at the G0/G1 or G2/M transition, they were deprived of serum for 48 h or treated with 200 ng/mL nocodazole (Sigma, St. Louis, MO, USA) for 20 h according to the previous reports 18,19. Cell-cycle analysis was performed as described previously 14–17.

### Tumorigenesis and tumoral growth in vivo

To investigate whether ANGPTL3 expression contributed to tumorigenesis and tumoral growth, we used xenograft models in two cell lines (HSC-3 and Sa3). The cells (2 × 10^7^) were independently injected subcutaneously into the backs of female nude mice, BALB/c-nu, purchased from Oriental Yeast Co. (Tokyo, Japan). All experimental animals were treated and cared for in accordance with institutional guidelines. The tumoral sizes were measured using a digital caliper every 3–4 days after injection, when the volume of the transplantation tumor reached 100 mm^3^. We used the formula 4*π*/3 × (width/2)^2^ × (length/2) to calculate tumoral volume. The mice were sacrificed after 28 days. Tumor tissues were fixed in 10% formalin, and paraffin sections (4 *μ*m) were prepared for hematoxylin and eosin staining and IHC of anti-ANGPTL3, anti-ERK1/2, anti-pERK1/2, and anti-Ki-67 (Santa Cruz Biotechnology).

### Statistical analysis

In comparisons of ANGPTL3 expression levels, statistical significance was evaluated using the Mann–Whitney *U*-test. Relationships between ANGPTL3 IHC staining scores and clinicopathological profiles were evaluated using the *χ*^2^ test, Fisher's exact test, and Mann–Whitney *U*-test. Survival curves were obtained using the Kaplan–Meier method, and differences in survival rates between ANGPTL3-positive and -negative cases were compared using the log-rank test. *P* < 0.05 was considered significant. The data are expressed as the mean ± standard error of the mean (SEM). We then performed the receiver operating characteristic (ROC) curve analysis by plotting sensitivity versus specificity, and area under the ROC curve (AUC) values with estimate odds ratios and 95% confidence intervals (CIs) as described previously with a slight modification 20. The identified data were expressed as the mean ± SEM. Statistical analyses were performed using EZR (Saitama Medical Center, Jichi Medical University, accessed 1 March 2012, at http://www.jichi.ac.jp/saitama-sct/SaitamaHP.files/statmedENstatmedEN.html), which is a graphical user interface for R (The R Foundation for Statistical Computing, version 2.13.0) 21.

## Results

### Upregulation of ANGPTL3 in OSCC-derived cell lines

**Figure 1:**
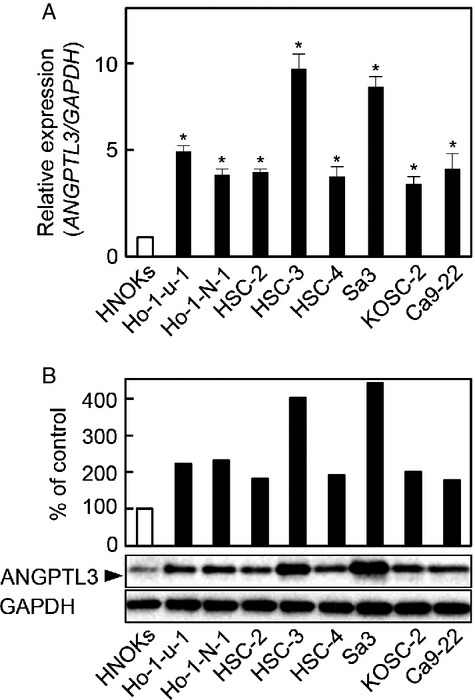
Evaluation of ANGPTL3 expression in OSCC-derived cell lines. (A) Quantification of *ANGPTL3* mRNA expression in OSCC-derived cell lines by qRT-PCR analysis. Significant (*P <* *0.05, Mann–Whitney *U*-test) upregulation of *ANGPTL3* mRNA is seen in seven OSCC-derived cell lines compared with the HNOKs. Data are expressed as the mean ± SEM of triplicate results. (B) Immunoblotting analysis of ANGPTL3 protein in the OSCC-derived cell lines and HNOKs. ANGPTL3 protein expression is upregulated in the OSCC-derived cell lines compared with the HNOKs. Densitometric ANGPTL3 protein data are normalized to GAPDH protein levels. The values are expressed as a percentage of the HNOKs (*P *<* *0.05, Mann–Whitney *U*-test).

To investigate the expression status of *ANGPTL3*, we performed qRT-PCR and immunoblotting analyses using eight OSCC-derived cell lines (Ho-1-N-1, Ho-1-u-1, HSC-2, HSC-3, HSC-4, Sa3, KOSC-2, and Ca9-22) and HNOKs. *ANGPTL3* mRNA was upregulated significantly (*P *<* *0.05) in all OSCC-derived cell lines compared with the HNOKs (Fig.1A). Representative results of immunoblotting analysis are shown in Figure1B. The ANGPTL3 protein expression decreased significantly (*P *<* *0.05) in all OSCC-derived cell lines compared with the HNOKs.

### Evaluation of ANGPTL3 expression in primary OSCCs

**Figure 2:**
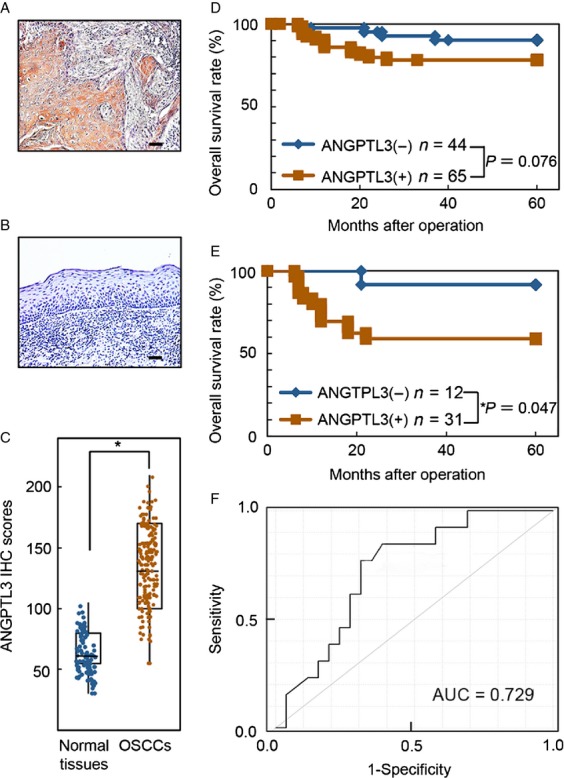
Evaluation of ANGPTL3 protein expression in primary OSCCs. (A and B) Representative IHC results of ANGPTL3 in primary OSCCs and normal oral tissues. Original magnification, ×100. Scale bars, 50 *μ*m. ANGPTL3 is highly overexpressed in OSCCs compared to normal oral tissues. (C) The status of ANGPTL3 protein expression in primary OSCCs (*n* = 109) and normal counterparts based on an IHC scoring system. IHC scores are calculated as follows: IHC score = 1 × (number of weakly stained cells in the field) + 2 × (number of moderately stained cells in the field) + 3 × (number of intensely stained cells in the field). The ANGPTL3 IHC scores for normal oral tissues and OSCCs range from 30.5 to 105.2 (median, 65.5) and 55.5 to 200.0 (median, 138.6), respectively. ANGPTL3 protein expression levels in OSCCs are significantly higher than in normal oral tissues (*P = 0.003; Mann–Whitney *U* test). (D) Kaplan–Meier curve for overall survival. The ANGPTL3 expression level is not correlated significantly (*P* = 0.076, log-rank test) with the overall survival. The overall survival rates in the ANGPTL3-positive OSCCs (*n* = 65) and the ANGPTL3-negative OSCCs (*n* = 44) were 78.7% and 89.9%, respectively. (E) Kaplan–Meier overall survival curves of patients with OSCC with a primary tumor size of T3/T4. A significant (*P* = 0.047, log-rank test) difference is seen in the overall survival rates between the ANGPTL3-positive OSCCs (*n* = 31, 58.6%) and the ANGPTL3-negative OSCCs (*n* = 12, 91.6%). (F) To evaluate the diagnostic relevance of the identified IHC scores, we used the ROC curve by plotting sensitivity versus specificity. The ANGPTL3 IHC scores of patients with primary T3/T4 OSCC tumors in primary OSCCs normalized to those in normal tissues. The optimal threshold value was 2.25 (sensitivity, 75.8%; specificity, 68.2%). When the cutoff values for the ANGPTL3 IHC scores were set at 2.25, the AUC was 0.7293 (95% CI, 0.5682–0.8903, *P *<* *0.05).

We analyzed the ANGPTL3 protein expression in primary OSCCs from 109 patients using an IHC scoring system. The ANGPTL3 IHC scores in OSCCs and adjacent normal oral tissues ranged from 55.5 to 200.0 (median, 138.6) and 30.5 to 105.2 (median, 65.5), respectively. Representative IHC results for ANGPTL3 protein in adjacent normal oral tissue and primary OSCCs are shown in Figure2A and B, respectively. Strong ANGPTL3 immunoreactivity was detected in the cytoplasmic of OSCC tissues, whereas the normal tissues showed almost negative immunostaining. The IHC scores in primary OSCCs were significantly (*P *<* *0.05) higher than in normal oral tissues (Fig.2C). The correlations between the clinicopathological characteristics of the patients with OSCC and the status of ANGPTL3 protein expression are shown in Table1. Among the clinical parameters, the ANGPTL3 expression level was significantly (*P *<* *0.05) related to the primary tumoral size of the OSCCs. Survival analysis using the Kaplan–Meier method showed that the ANGPTL3 expression level was not significantly (*P *=* *0.076) correlated with overall survival (Fig.2D). The overall survival rates in the ANGPTL3-positive OSCCs (*n* = 65) and the ANGPTL3-negative OSCCs (*n* = 44) were 78.7% and 89.9%, respectively. However, a significant (*P *=* *0.047) difference was found in overall survival rates of patients with primary T3/T4 OSCC tumors between the ANGPTL3-positive OSCCs (*n* = 31; 58.6%) and the ANGPTL3-negative OSCCs (*n* = 12; 91.6%) (Fig.2E). Furthermore, the diagnostic accuracy of the identified IHC scores was assessed using the ROC curve analysis. The ANGPTL3 IHC scores of patients with primary T3/T4 OSCC tumors in primary OSCCs normalized to those in normal tissues. The optimal threshold value was 2.25 (sensitivity, 76.9%; specificity, 69.2%). When the cutoff values for the ANGPTL3 IHC scores were set at 2.25, the AUC was 0.7293 (95% CI, 0.5682–0.8903, *P *<* *0.05) (Fig.2F).

**Table 1:** Correlation between ANGPTL3 expression and clinical classification in OSCCs

Clinical classification		Results of immunostaining no. patients (%)		
	Total	ANGPTL3 negative	ANGPTL3 positive	*P* value^1^
Age at surgery (years)				
<60	30	13 (43)	17 (57)	0.36^2^
≥60, <70	26	13 (50)	13 (50)	
≥70	53	18 (32)	35 (68)	
Gender				
Male	74	31 (42)	43 (58)	0.68^3^
Female	35	13 (36)	22 (64)	
T-primary tumor				
T1	8	6 (75)	2 (25)	0.01^4^
T2	58	26 (44)	32 (56)	
T3	20	6 (30)	14 (70)	
T4	23	6 (26)	17 (74)	
T1 + T2	66	32 (43)	34 (57)	0.03^3^
T3 + T4	43	12 (27)	31 (73)	
N-regional lymph node				
N (−)	63	26 (43)	37 (58)	0.82^3^
N (+)	46	18 (39)	28 (61)	
Stage				
I	7	5 (71)	2 (29)	0.06^4^
II	45	19 (43)	26 (57)	
III	18	9 (50)	9 (50)	
IV	39	11 (28)	28 (72)	
Vascular invasion				
V(−)	79	32 (40)	47 (60)	0.96^3^
V(+)	30	12 (40)	18 (60)	
Histopathologic type				
Well	61	24 (38)	37 (65)	0.78^4^
Moderately	42	17 (40)	25 (60)	
Poorly	6	3 (50)	3 (50)	
Tumoral site				
Gingiva	35	10 (34)	25 (66)	0.19^4^
Tongue	61	29 (52)	32 (48)	
Buccal mucosa	7	3 (42)	4 (58)	
Oral floor	6	2 (33)	4 (67)	
^ ^Total	109	44 (40)	65 (60)	

1 *P *< 0.05 was considered significant.

2 *χ*^2^ test.

3 Fisher's exact test.

4 Mann–Whitney's *U*-test.

### Establishment of ANGPTL3 knockdown cells

**Figure 3:**
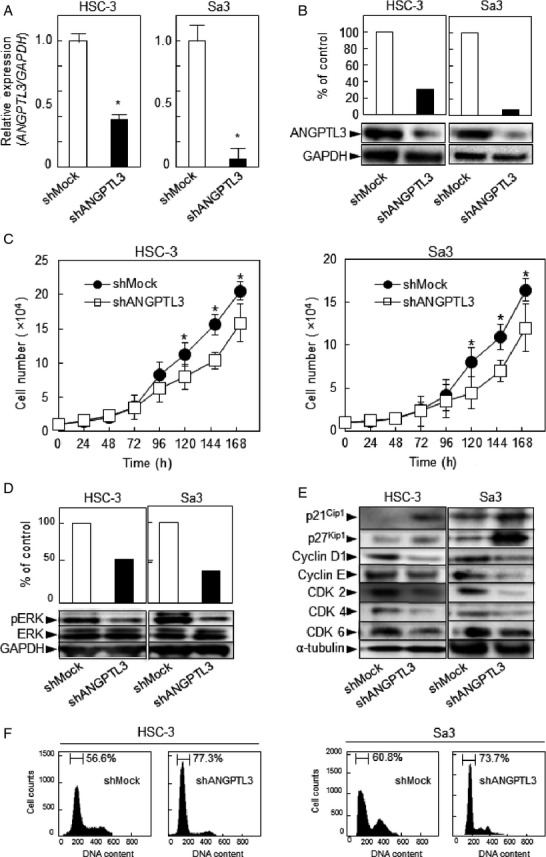
Expression of ANGPTL3 and ANGPTL3 knockdown inhibits ERK activation and promotes G1 arrest. (A) qRT-PCR shows that *ANGPTL3* mRNA expression in the shANGPTL3-transfected cells (HSC-3- and Sa3-derived transfectants) is significantly (*P <* *0.05, Mann–Whitney *U*-test) lower than in the shMock-transfected cells. (B) Immunoblotting analysis shows that the ANGPTL3 protein levels in the shANGPTL3-transfected cells (HSC-3- and Sa3-derived transfectants) also are decreased markedly compared with the shMock-transfected cells. (C) To determine the effect of shANGPTL3 on cellular proliferation, shANGPTL3-, and shMock-transfected cells were seeded in six-well plates at a density of 1 × 10^4^ viable cells/well. Both transfectants were counted on seven consecutive days. The cellular growth of shANGPTL3-transfected cells (HSC-3- and Sa3-derived transfectants) is significantly inhibited compared with the shMock-transfected cells after 7 days (168 h). The results are expressed as the means ± SEM of values from three assays. The asterisks indicate significant (*P <* *0.05, Mann–Whitney *U*-test) differences between the shANGPTL3 and shMock cells. (D) Immunoblotting analysis shows that ANGPTL3 knockdown results in decreased levels of pERK compared with the shMock-transfected cells (HSC-3- and Sa3-derived transfectants). Densitometric pERK/ERK protein data are normalized to GAPDH protein levels. (E) Immunoblotting analysis shows upregulation of p21^Cip1^ and p27^Kip1^ and downregulation of cyclin D1, cyclin E, CDK2, CDK4, and CDK6 in the shANGPTL3-transfected cells (HSC-3- and Sa3-derived transfectants) compared with the shMock-transfected cells. (F) Flow cytometric analysis was performed to investigate cell-cycle progression in the shANGPTL3- and shMock-transfected cells after synchronization at the G2/M phase to treatment with nocodazole. The percentage of cells at the G1 phase in the shANGPTL3-transfected cells (HSC-3- and Sa3-derived transfectants) is increased markedly compared with the shMock- transfected cells.

Since frequent upregulation of ANGPTL3 was observed in OSCC-derived cells (Fig.1), the OSCC-derived cells (HSC-3 and Sa3) were transfected with ANGPTL3 shRNA and shMock as controls. To confirm that shANGPTL3 transfection works and ANGPTL3 mRNA and protein decrease, we performed qRT-PCR and Western blotting (Fig.3A and B, respectively). The *ANGPTL3* mRNA expression in shANGPTL3 cells was significantly (*P *<* *0.05) lower than in shMock cells (Fig.3A). The ANGPTL3 protein level in the shANGPTL3 cells also was decreased compared with shMock cells (Fig.3B).

### Functional analyses of ANGPTL3 knockdown cells

We performed a cellular proliferation assay (Fig.3C) to evaluate the effect of ANGPTL3 knockdown on cellular growth, we found a significant (*P *<* *0.05) decrease in cellular growth in all shANGPTL3 cells compared with shMock cells. The assays showed that ANGPTL3 knockdown decreased cellular growth.

### Inactivation of the ERK pathway in shANGPTL3 cells

We assessed the phosphorylation level of ERK in shANGPTL3 by immunoblotting analysis. The level of phosphorylated ERK (pERK) protein decreased significantly in shANGPTL3 cells compared with shMock cells (Fig.3D). Furthermore, to evaluate the effect of ERK inhibitor, we performed immunoblotting analysis with 10 *μ*mol/L PD184352 (Sigma) or an equivalent 0.1% DMSO (control) for 90 min 22,23. Results from immunoblotting analysis indicated the similar results in shANGPTL3 cells (Fig. S1A). These results suggested that the ERK signaling pathway was attenuated frequently in the shANGPTL3 cells.

### Cell-cycle analysis of shANGPTL3 cells

We assessed the expression levels of the cyclin-dependent kinase inhibitors (CDKIs) (p21^Cip1^ and p27^Kip1^), cyclins, and CDKs. As expected, the CDKIs were upregulated, and cyclin D1, cyclin E, CDK2, CDK4, and CDK6 were significantly (*P *<* *0.05) downregulated in the shANGPTL3 cells (Fig.3E). Moreover, the percentage of the shANGPTL3 cells at the G1 phase was significantly (*P *<* *0.05) higher than of the Mock cells (Fig.3F). These results indicated that shANGPTL3 inhibited cellular proliferation by cell-cycle arrest at the G1 phase.

### ANGPTL3 promoted tumoral growth in vivo

**Figure 4:**
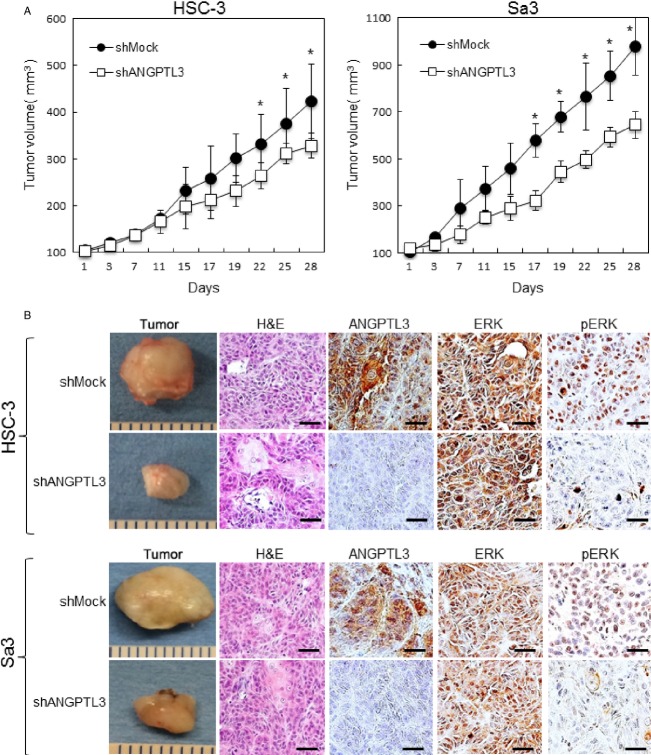
ANGPTL3 promotes tumoral growth in vivo. (A) ShANGPTL3- and shMock-transfected cells (HSC-3 and Sa3) were injected subcutaneously into the backs of female nude mice (*n* = 3). Tumoral growth in the shANGTPL3-injected mice is inhibited significantly (*P <* *0.05; Mann–Whitney *U*-test) compared to the shMock-injected mice. (B) IHC of the xenografted tumors clearly shows more decreased immunostaining for ANGPTL3 and pERK in the xenografted tumors from shANGPTL3 transfectants than shMock transfectants. H&E staining confirmed the presence of tumoral cells. Original magnification, ×400. Scale bars, 50 *μ*m.

We assessed the effect of ANGPTL3 on tumoral growth in vivo by evaluating to target tumor xenografts in nude mice. shANGPTL3- and shMock-transfected cells of two cell lines, HSC-3 and Sa3 were injected subcutaneously into the backs of female nude mice, respectively (three mice in each group). According to our in vitro findings, the mean tumoral volume of the shANGPTL3-transfected cells was significantly (*P *<* *0.05) smaller than that of the shMock-transfected cells (Fig.4A). ANGPTL3 IHC of tumoral sections showed that ANGPTL3 knockdown was maintained in vivo. Xenografted tumors of ANGPTL3 knockdown cells showed a significant decrease in the pERK and Ki-67 levels (Fig.4B and Fig. S1B). However, the ERK levels were unchanged. These study provided that ANGPTL3 promotes tumoral growth by the ERK pathway in nude mice.

## Discussion

We showed that ANGPTL3 was overexpressed frequently in all OSCC-derived cell lines examined (Fig.1). The knockdown of ANGPTL3 in OSCC-derived cell lines resulted in a dramatic effect on growth inhibition in vitro and in vivo via arrest of the G1/S phase by inactivation of ERK with upregulation of CDKI including p21^Cip1^ and p 27^Kip1^, suggesting that ANGPTL3 may predict tumoral progression and be a prognostic factor of this disease. (Figs.3 and 4).

The expression and function of ANGTPL3 in OSCCs have not been investigated previously. In this study, we found that ANGPTL3 was upregulated at the pretranscription and protein levels in all OSCC-derived cell lines examined (Fig.1). To investigate the possible function of ANGPTL3, we created stable ANGPTL3 knockdown transfectants in two OSCC-derived cell lines (Fig.3A and B). Interestingly, suppression of ANGPTL3 significantly decreased cellular proliferation (Fig.3C), which suggested the hypothesis that ANGPTL3 expression is necessary for oral carcinogenesis and neoplastic progression.

It is thought that carcinogenesis of human cancers including OSCC are multistep processes with epithelial cellular proliferation of progressive disruption. The study of cellular proliferation mechanism in tumoral cells is important for tumoral treatment and molecular biology 24,25. ANGPTL3 has been reported to be associated with tumoral development and progression 26. In this study, suppression of ANGPTL3 by shRNA significantly decreased cellular proliferation by inhibiting the ERK pathway (Fig.3D). Knockdown of ANGPTL3 is related closely to hyperactivation of the ERK signaling pathway, which is linked to fundamental cellular processes such as cell-cycle arrest and proliferation 27,28. In addition, extracellular signals tightly control the ERK pathway 29–31, which include the ANGPTL3 binding to integrin *α*_γ_*β*3 1,7,32. Based on this evidence combined with the current results, we believe that ANGPTL3 may be correlated at least indirectly with aberrant activation of ERK/MAPK signaling pathways.

More importantly, our OSCC cells with shANGPTL3 have the characteristics to arrest with upregulation of p21^Cip1^ and p 27^Kip1^ and downregulation of cyclin D1 and cyclin E at the G1 phase of cell cycle (Fig.3E). Considerable evidence has shown that while cyclin D1 and cyclin E are upregulated 14,33, the CDKIs including p21^Cip1^ and p 27^Kip1^ are downregulated in human oral cancers 15,16. In addition, G1-S transition of cell cycle is blocked by dramatic inhibition of their expression 9,34,35. Members of the Cip/Kip family, p21^Cip1^ and p27^Kip1^, are implicated in the negative regulation of cell-cycle progression from the G1 to S phase by binding to and modulating CDK activity 8,36–39. In contrast, CDK complexes promote progression from the G1 to S phase by triggering DNA replication and regulating genes 35,40,41. ERK regulates p21^Cip1^ and p27^Kip1^ negatively and CDK complexes positively 35,40,41. These data suggested that ANGPTL3 regulates the ERK signaling pathway and promotes the G1 cell cycle during OSCC progression.

Moreover, to investigate the oncogenic potential of ANGPTL3 in vivo, we tested a xenograft assay. Our in vivo data showed significant inhibition of xenografted tumoral growth and transformation, and activation of ERK in tumoral tissues by ANGPTL3 silencing. Therefore, our current study suggested that ANGPTL3 activates the MAPK signaling pathway via pERK and promotes the G1 cell cycle by downregulating CDKIs during OSCC progression.

Based on our in vivo and in vitro data, we concluded that aberrant ANGPTL3 expression in primary OSCCs may contribute to neoplastic promote via the ERK pathway that ANGPTL3 may play an important role in OSCC cellular growth. It would be interesting to design clinical studies to elucidate the predictive relevance of ANGPTL3 expression status in future patients with OSCC, because advanced OSCC cases (T3/T4) have been divided into two groups, those with favorable and unfavorable prognoses (Fig.2 E and F), based on the ANGPTL3 status in primary OSCCs.

## Supporting Information

**Figure S1.** PD184352 blocks activation of ERK and proliferation maker Ki-67 of xenografted tumors. (A) Immunoblotting analysis shows that PD184352 treatment results in decreased levels of pERK compared with the control cells. (B) IHC of the xenografted tumors clearly shows more decreased immunostaining for Ki-67 in the xenografted tumors from shANGPTL3 transfectants than shMock transfectants. Original magnification, ×400. Scale bars, 50 *μ*m.

## References

[b1] Camenisch G, Pisabarro MT, Sherman D, Kowalski J, Nagel M, Hass P (2002). ANGPTL3 stimulates endothelial cell adhesion and migration via integrin *α*v*β*3 and induces blood vessel formation in vivo. J. Biol. Chem.

[b2] Li Y, Teng C (2014). Angiopoietin-like proteins 3, 4 and 8: regulating lipid metabolism and providing new hope for metabolic syndrome. J. Drug Target.

[b3] Ono M, Shimizugawa T, Shimamura M, Yoshida K, Noji-Sakikawa C, Ando Y (2003). Protein region important for regulation of lipid metabolism in angiopoietin-like 3 (ANGPTL3) ANGPTL3 IS CLEAVED AND ACTIVATED IN VIVO. J. Biol. Chem.

[b4] Yu H, Zhang H, Li D, Xue H, Pan C, Zhao S (2011). Effects of ANGPTL3 antisense oligodeoxynucleotides transfection on the cell growths and invasion of human hepatocellular carcinoma cells. Hepatogastroenterology.

[b5] Chambard J-C, Lefloch R, Pouysségur J, Lenormand P (2007). ERK implication in cell cycle regulation. Biochim. Biophys. Acta (BBA)-Mol. Cell Res.

[b6] Massagué J (2004). G1 cell-cycle control and cancer. Nature.

[b7] Broxmeyer HE, Srour EF, Cooper S, Wallace CT, Hangoc G, Youn BS (2012). Angiopoietin-like-2 and -3 act through their coiled-coil domains to enhance survival and replating capacity of human cord. Blood hematopoietic progenitors. Blood Cells Mol. Dis.

[b8] Mebratu Y, Tesfaigzi Y (2009). How ERK1/2 activation controls cell proliferation and cell death: is subcellular localization the answer?. Cell Cycle (Georgetown, Tex).

[b9] Meloche S, Pouyssegur J (2007). The ERK1/2 mitogen-activated protein kinase pathway as a master regulator of the G1- to S-phase transition. Oncogene.

[b10] Albanese C, Johnson J, Watanabe G, Eklund N, Vu D, Arnold A (1995). Transforming p21ras mutants and c-Ets-2 activate the cyclin D1 promoter through distinguishable regions. J. Biol. Chem.

[b11] Lavoie JN, L'Allemain G, Brunet A, Muller R, Pouyssegur J (1996). Cyclin D1 expression is regulated positively by the p42/p44MAPK and negatively by the p38/HOGMAPK pathway. J. Biol. Chem.

[b12] Greulich H, Erikson RL (1998). An analysis of Mek1 signaling in cell proliferation and transformation. J. Biol. Chem.

[b13] Bhatt KV, Spofford LS, Aram G, McMullen M, Pumiglia K, Aplin AE (2005). Adhesion control of cyclin D1 and p27Kip1 levels is deregulated in melanoma cells through BRAF-MEK-ERK signaling. Oncogene.

[b14] Shimizu T, Kasamatsu A, Yamamoto A, Koike K, Ishige S, Takatori H (2012). Annexin A10 in human oral cancer: biomarker for tumoral growth via G1/S transition by targeting MAPK signaling pathways. PLoS One.

[b15] Iyoda M, Kasamatsu A, Ishigami T, Nakashima D, Endo-Sakamoto Y, Ogawara K (2010). Epithelial cell transforming sequence 2 in human oral cancer. PLoS ONE.

[b16] Baba T, Sakamoto Y, Kasamatsu A, Minakawa Y, Yokota S, Higo M (2013). Persephin: a potential key component in human oral cancer progression through the RET receptor tyrosine kinase-mitogen-activated protein kinase signaling pathway. Mol. Carcinogen.

[b17] Shimizu F, Shiiba M, Ogawara K, Kimura R, Minakawa Y, Baba T (2013). Overexpression of LIM and SH3 protein 1 leading to accelerated G2/M phase transition contributes to enhanced tumourigenesis in oral cancer. PLoS One.

[b18] Ferrero M, Ferragud J, Orlando L, Valero L, del Pino MS, Farràs R (2011). Phosphorylation of AIB1 at mitosis is regulated by CDK1/CYCLIN B. PLoS ONE.

[b19] Díaz-Rodríguez E, Álvarez-Fernández S, Chen X, Paiva B, López-Pérez R, García-Hernández JL (2011). Deficient spindle assembly checkpoint in multiple myeloma. PLoS ONE.

[b20] Uzawa K, Baba T, Uchida F, Yamatoji M, Kasamatsu A, Sakamoto Y (2012). Circulating tumor-derived mutant mitochondrial DNA: a predictive biomarker of clinical prognosis in human squamous cell carcinoma. Oncotarget.

[b21] Kanda Y (2013). Investigation of the freely available easy-to-use software ‘EZR’ for medical statistics. Bone Marrow Transplant.

[b22] Squires M, Nixon P, Cook S (2002). Cell-cycle arrest by PD184352 requires inhibition of extracellular signal-regulated kinases (ERK) 1/2 but not ERK5/BMK1. Biochem. J.

[b23] Sebolt-Leopold JS, Dudley DT, Herrera R, Van Becelaere K, Wiland A, Gowan RC (1999). Blockade of the MAP kinase pathway suppresses growth of colon tumors in vivo. Nat. Med.

[b24] Fearon ER, Vogelstein B (1990). A genetic model for colorectal tumorigenesis. Cell.

[b25] Marshall CJ (1991). Tumor suppressor genes. Cell.

[b26] Yu H, Zhang H, Li D, Xue H, Pan C, Zhao S (2010). Effects of ANGPTL3 antisense oligodeoxynucleotides transfection on the cell growths and invasion of human hepatocellular carcinoma cells. Hepatogastroenterology.

[b27] Coolican SA, Samuel DS, Ewton DZ, McWade FJ, Florini JR (1997). The mitogenic and myogenic actions of insulin-like growth factors utilize distinct signaling pathways. J. Biol. Chem.

[b28] McCubrey JA, Steelman LS, Franklin RA, Abrams SL, Chappell WH, Wong EW (2007). Targeting the RAF/MEK/ERK, PI3K/AKT and p53 pathways in hematopoietic drug resistance. Adv. Enzyme Regul.

[b29] Orton RJ, Sturm OE, Vyshemirsky V, Calder M, Gilbert DR, Kolch W (2005). Computational modelling of the receptor-tyrosine-kinase-activated MAPK pathway. Biochem. J.

[b30] Giancotti FG, Ruoslahti E (1999). Integrin signaling. Science.

[b31] Dhillon A, Hagan S, Rath O, Kolch W (2007). MAP kinase signalling pathways in cancer. Oncogene.

[b32] Giancotti FG (2000). Complexity and specificity of integrin signalling. Nat. Cell Biol.

[b33] Uchida F, Uzawa K, Kasamatsu A, Takatori H, Sakamoto Y, Ogawara K (2013). Overexpression of CDCA2 in human squamous cell carcinoma: correlation with prevention of G1 phase arrest and apoptosis. PLoS ONE.

[b34] Viglietto G, Motti ML, Fresco A (2002). Understanding p27kip1 deregulation in cancer: downregulation or mislocalizaiton?. Cell Cycle (Georgetown, Tex).

[b35] Perisanidis C, Perisanidis B, Wrba F, Brandstetter A, El Gazzar S, Papadogeorgakis N (2012). Evaluation of immunohistochemical expression of p53, p21, p27, cyclin D1, and Ki67 in oral and oropharyngeal squamous cell carcinoma. J. Oral Pathol. Med.

[b36] Kisielewska J, Philipova R, Huang JY, Whitaker M (2009). MAP kinase dependent cyclinE/cdk2 activity promotes DNA replication in early sea urchin embryos. Dev. Biol.

[b37] Wey JS, Gray MJ, Fan F, Belcheva A, McCarty MF, Stoeltzing O (2005). Overexpression of neuropilin-1 promotes constitutive MAPK signalling and chemoresistance in pancreatic cancer cells. Br. J. Cancer.

[b38] Mountzios G, Planchard D, Besse B, Validire P, Girard P, Devisme C (2008). Mitogen-activated protein kinase activation in lung adenocarcinoma: a comparative study between ever smokers and never smokers. Clin. Cancer Res.

[b39] Shrestha Y, Schafer EJ, Boehm JS, Thomas SR, He F, Du J (2012). PAK1 is a breast cancer oncogene that coordinately activates MAPK and MET signaling. Oncogene.

[b40] Musgrove EA, Lee CS, Buckley MF, Sutherland RL (1994). Cyclin D1 induction in breast cancer cells shortens G1 and is sufficient for cells arrested in G1 to complete the cell cycle. Proc. Natl. Acad. Sci. USA.

[b41] Tam SW, Theodoras AM, Shay JW, Draetta GF, Pagano M (1994). Differential expression and regulation of cyclin D1 protein in normal and tumor human cells: association with Cdk4 is required for cyclin D1 function in G1 progression. Oncogene.

