# Supplementary material for: ANGPTL3 is a novel biomarker as it activates ERK/MAPK pathway in oral cancer
Source: Cancer Med. 2015 Jan 30;4(5):759–69. doi: 10.1002/cam4.418 (PMC4430268; doi:10.1002/cam4.418)
Supplement: Supplementary file 1 — Figure S1. PD184352 blocks activation of ERK and proliferation maker Ki-67 of xenografted tumors. (A) Immunoblotting analysis shows that PD184352 treatment results in decreased levels of pERK compared with the control cells. (B) IHC of the xenografted tumors clearly shows more decreased immunostaining for Ki-67 in the xenografted tumors from shANGPTL3 transfectants than shMock transfectants. Original magnification, ×400. Scale bars, 50 μm. [file cam40004-0759-sd1.docx]

Figure S1.

PD184352 blocks activation of ERK and proliferation maker Ki-67 of xenografted tumors. (A) Immunoblotting analysis shows that PD184352 treatment results in decreased levels of pERK compared with the control cells. (B) IHC of the xenografted tumors clearly shows more decreased immunostaining for Ki-67 in the xenografted tumors from shANGPTL3-transfectants than shMock-transfectants. Original magnification, ×400. Scale bars, 50 μm.
